# Regenerative Neurology and Regenerative Cardiology: Shared Hurdles and Achievements

**DOI:** 10.3390/ijms23020855

**Published:** 2022-01-13

**Authors:** Dinko Mitrečić, Valentina Hribljan, Denis Jagečić, Jasmina Isaković, Federica Lamberto, Alex Horánszky, Melinda Zana, Gabor Foldes, Barbara Zavan, Augustas Pivoriūnas, Salvador Martinez, Letizia Mazzini, Lidija Radenovic, Jelena Milasin, Juan Carlos Chachques, Leonora Buzanska, Min Suk Song, András Dinnyés

**Affiliations:** 1 Laboratory for Stem Cells, Croatian Institute for Brain Research, University of Zagreb School of Medicine, 10000 Zagreb, Croatia; valentina.hribljan@mef.hr (V.H.); denis.jagecic@mef.hr (D.J.); 2 Department of Histology and Embryology, University of Zagreb School of Medicine, 10000 Zagreb, Croatia; 3 Omnion Research International Ltd., 10000 Zagreb, Croatia; jasmina.isakovic@omnion-research.com (J.I.); min.suk.song@omnion-research.com (M.S.S.); 4 BioTalentum Ltd., Aulich Lajos Str. 26, 2100 Gordillo, Hungary; federica.lamberto@biotalentum.hu (F.L.); alex.horanszky@biotalentum.hu (A.H.); melinda.zana@biotalentum.hu (M.Z.); andras.dinnyes@biotalentum.hu (A.D.); 5 Department of Physiology and Animal Health, Institute of Physiology and Animal Health, Hungarian University of Agriculture and Life Sciences, Páter Károly Str. 1, 2100 Godollo, Hungary; 6 Heart and Vascular Center, Semmelweis University, 1122 Budapest, Hungary; foldes.gabor@med.semmelweis-univ.hu; 7 National Heart and Lung Institute, Imperial College London, London W12 0NN, UK; 8 Department of Translational Medicine, University of Ferrara, 44121 Ferrara, Italy; barbara.zavan@unipd.it; 9 Department of Stem Cell Biology, State Research Institute Centre for Innovative Medicine, LT-01102 Vilnius, Lithuania; augustas.pivoriunas@imcentras.lt; 10 Instituto de Neurociencias UMH-CSIC, 03550 San Juan de Alicante, Spain; smartinez@umh.es; 11 ALS Center, Department of Neurology, Maggiore della Carità Hospital, University of Piemonte Orientale, 28100 Novara, Italy; letizia.mazzini@uniupo.it; 12 Center for Laser Microscopy, Faculty of Biology, University of Belgrade, 11000 Belgrade, Serbia; lidijar@bio.bg.ac.rs; 13 Laboratory for Stem Cell Research, School of Dental Medicine, University of Belgrade, 11000 Belgrade, Serbia; jelena.milasin@stomf.bg.ac.rs; 14 Laboratory of Biosurgical Research, Pompidou Hospital, University of Paris, 75006 Paris, France; j.chachques-ext@aphp.fr; 15 Department of Stem Cell Bioengineering, Mossakowski Medical Research Institute Polish Academy of Sciences, 02-106 Warsaw, Poland; buzanska@imdik.pan.pl; 16 HCEMM-USZ Stem Cell Research Group, Department of Cell Biology and Molecular Medicine, University of Szeged, 6720 Szeged, Hungary; 17 College of Life Sciences, Sichuan University, Chengdu 610064, China

**Keywords:** stem cells, regenerative neuroscience, brain regeneration, neurology, cardiology, myocardial regeneration, clinical trials

## Abstract

From the first success in cultivation of cells in vitro, it became clear that developing cell and/or tissue specific cultures would open a myriad of new opportunities for medical research. Expertise in various in vitro models has been developing over decades, so nowadays we benefit from highly specific in vitro systems imitating every organ of the human body. Moreover, obtaining sufficient number of standardized cells allows for cell transplantation approach with the goal of improving the regeneration of injured/disease affected tissue. However, different cell types bring different needs and place various types of hurdles on the path of regenerative neurology and regenerative cardiology. In this review, written by European experts gathered in Cost European action dedicated to neurology and cardiology-Bioneca, we present the experience acquired by working on two rather different organs: the brain and the heart. When taken into account that diseases of these two organs, mostly ischemic in their nature (stroke and heart infarction), bring by far the largest burden of the medical systems around Europe, it is not surprising that in vitro models of nervous and heart muscle tissue were in the focus of biomedical research in the last decades. In this review we describe and discuss hurdles which still impair further progress of regenerative neurology and cardiology and we detect those ones which are common to both fields and some, which are field-specific. With the goal to elucidate strategies which might be shared between regenerative neurology and cardiology we discuss methodological solutions which can help each of the fields to accelerate their development.

## 1. Introduction

Regenerative medicine aims at replacing human cells, tissues or organs damaged by disease or aging and restoring their normal functions. Among some of the most promising approaches in regenerative medicine are stem cell-based therapies, which may provide unparalleled possibilities in the treatment of various conditions, including brain and heart diseases [1]. The therapeutic effect, i.e., restoration of function in the damaged tissue, is attained through direct cell replacement, stimulation of endogenous regeneration/repair systems, establishment of a supportive environment for the remaining cells, or a combination of these mechanisms [2]. Besides the progenitor cells from the affected nervous or cardiac muscle tissue, stem cells of most diverse origins have also proved to be candidates with great potential for translation towards clinical trials. Human embryonic stem cells (hESCs) show the highest differentiation potential, yet their use is hampered by numerous ethical controversies, and alternative sources are sought for [3]. Multipotency of mesenchymal stem cells (MSCs), their presence in almost all adult tissues, the fact that they are usually easily accessible and that they can be differentiated into neural and myocardial lineages makes them very appealing in cell-based therapies [4,5]. Lately, great expectations have also been placed on induced pluripotent stem cells (iPSCs) generated from somatic cells that have undergone genetic reprogramming resulting in pluripotency. Most of cell types are easily obtainable and expanded, usually with no need for immunosuppression following transplantation [6]. Over the past two decades a large number of trials have been conducted, and many are currently underway, including those related to demyelinating diseases and spinal cord injuries, amyotrophic lateral sclerosis, stroke, Parkinson’s disease, macular degeneration, as well as acute myocardial infarction, ischemic cardiomyopathy, refractory angina and many others [1]. Although different clinical trials in the fields of neurology and cardiology have reported promising benefits of stem cell-based therapies, many challenges still remain.

This review outlines various types of stem cells that are currently available in neurological and cardiovascular regenerative medicine and reports current state of the art in attempts to introduce those procedures in every day practice. Moreover, we analyse the signalling pathways and mechanisms of their action and examine the outcomes that have been reached with their application. In addition, we discuss the use of novel biomaterials as support for 2D and 3D cell growth, as well as the emerging role of exosomes and their cargos in tissue regeneration. Finally, an overview of the main obstacles, some shared between these two fields, and some field-specific, which we have yet to overcome are given.

## 2. Induced Pluripotent Stem Cells–Flying Start to Boosting In Vitro Models of the Nervous System and the Heart

**Figure 1:**
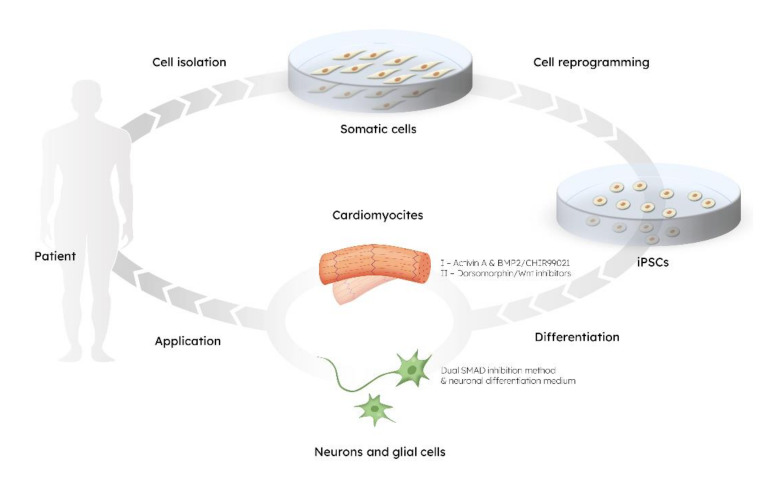
The circle of stem cells–based technology: from cell isolation to application.

The pioneer studies of Yamanaka and his group yielded protocols for obtaining induced pluripotent stem cells (iPSCs), thus providing the opportunity of dedifferentiating any cell to pluripotent state and, equally important, to obtain autologous, patient-specific cells [7,8]. IPSCs are generated from somatic cells, which have been reprogrammed to acquire pluripotency and have the unique capabilities of self-renewal, proliferation, and differentiation [9]. Since iPSCs can give rise to virtually any cellular lineage, an important application of iPSC technology is the in vitro differentiation of specialized cells, like neurons and cardiomyocytes (Figure 1). This can then be used for investigation of a specific tissue, including both fully differentiated cells and their precursors. Such an approach paved the way for promising advances in patient-specific disease modelling, drug screening, and cell-based therapies without the risk of immune rejection [9,10,11,12].

## 3. iPSC-Derived Cardiomyocytes

The course of cardiomyocytes differentiation is validated using molecular markers for different stages of development, as well as investigating their beating capacity, electrophysiology and metabolism [13,14,15]. Most importantly, we developed methods to differentiate iPSC in cardiomyocytes, with the goal to generate a mixed population of cardiac cells, as ventricular-, atrial- and pacemaker-like cardiomyocytes [16]. That protocol consists of two major steps: firstly, growth factors (e.g., activin A, BMP2) or small molecules (e.g., CHIR99021) activate the Wnt signalling, allowing the mesoderm (*Nkx2.5+*, *Gata4+, Mesp1+*) induction [14,17,18,19]. Secondly, small molecules (e.g., dorsomorphin) or Wnt inhibitors (e.g., IWR-1, IWP-2) are used to enhance the cardiac lineage specification and differentiation (*cTnT+*, *Myh6+*, *Tnni3+*) [17,19,20]. Under these conditions, ventricular-like cardiomyocytes (*Hey2*+, *Mlc2v*+) are predominant than other cardiac cell types [14,21,22,23]. However, depending on the research aim, there are also methods to purify and isolate an atrial-like cell population (*Kcnj3+, Kcnj5+*, *Cacna1d+*), for example using retinoic acid or BMP antagonist (e.g., Noggin, Gremlin 2), by upregulating atrial-specific genes. [14,23,24,25,26,27]. On the other hand, the pacemaker-like cardiomyocytes are still difficult to obtain *in vitro*. So far, the inhibition of neuregulin1β/ErbB signalling seems the most efficient way to enrich the sinoatrial node cells population (*Hcn4*+, *Tbx3*+, *Tbx18*+), and only recently it has been hypothesized that modulating the Wnt signalling through Nodal inhibition may promote the pacemaker cells fate [28,29,30].

## 4. Specific Requirements for In Vitro Heart Muscle Model

**Figure 2:**
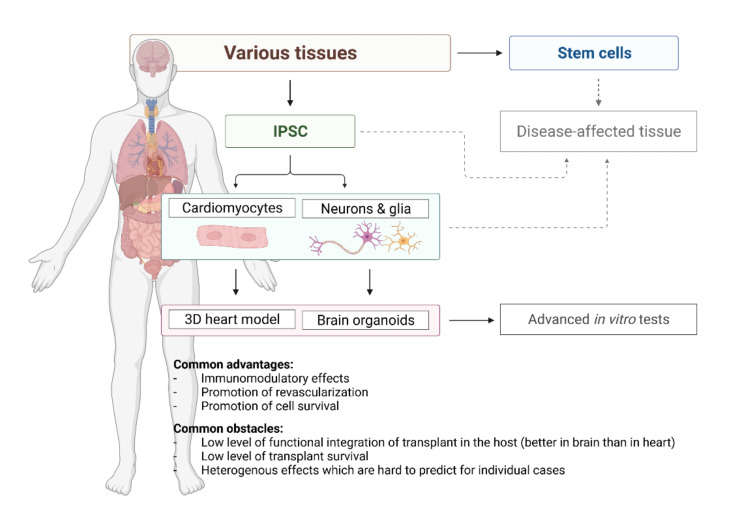
Current options offered by stem cell-based technology for regenerative cardiology and neurology.

The iPSC-derived cardiomyocytes correspond to the foetal-like state concerning their functional and physiological characteristics [31]. A very specific challenge is to obtain more mature cardiomyocytes, and several methods are currently available [32]. Compared to immature counterparts, these adult-like cardiomyocytes metabolise fatty acids, display a high mitochondrial mass, well-arranged sarcomeres, and higher contraction force. Therefore, cardiomyocytes maturation can be achieved by poviding fatty acids to the culture medium [33], using mechanical and electrical stimulation [34] or developing a 3D cellular model [21]. Interestingly, iPSCs obtained from cardiac sources suggest an improved differentiation capacity in vitro and possibly a higher degree of maturation of cardiomyocytes. In this regard, the epigenetic memory of somatic cell source may play a fundamental role [35,36]. When the current state of the art is taken in account, the most promising approach is cultivating cardiomyocytes in 3D form (Figure 2). It comes very close to heart’s unique cytoarchitectural arrangement and to an even higher level of similarity to the original tissue, with the ultimate goal to establish a heart-on-a-chip model [21,37]. This scaffold-based approach can mimic the patient-specific anatomical microstructure and composition of the human heart and vessels as well as generate responsive constructs to study intact tissue-level cardiovascular physiology. The interaction between cells and the cardiovascular extracellular niche and matrix constituents leads to activation of physiological underlying mechanisms and responsiveness to mechanical, electrical and pharmacological cues. Thus, multicellular microtissue may prove useful for many cell-based applications, like cardiotoxicity assessment and modelling myocardial infarction in a dish [38]. However, comparing structural, mechanical, and biological properties of these structures head-to-head with perfused intact tissues like myocardial and vascular slices and wedges is still warranted.

Another critical cell subtype of the cardiovascular system is those forming the organotypic vasculature [39]. The generation of these endothelial cells should also rely on organ-specific differentiation protocols, where functional readouts can validate the efficacy and quality of the production. Their specific function comprises barrier-forming continuous layers, a specific vasoactive and growth factor secretion profile and thrombogenic properties [39,40]. Most importantly, being more than a passive conduit, prevascularisation by these endothelial cells can support the long-term survival and instruct the contractility and other functions of adjacent cardiomyocytes within the in vitro generated multicellular constructs. To establish vascularisation, pluripotent stem cell-derived endothelial cells show a remarkable capacity to self-organise into functional microvasculature, like cardiac capillaries, thereby providing sufficient perfusion throughout the cell constructs with a substantial thickness [41].

## 5. iPSC-Derived Neurons

The human brain is comprised of a combination of distinct cellular subtypes with a diverse range of specialized functions such as electrical communication, axonal ensheating and metabolic coupling [42]. These include, but are not limited to neurons, which are the primary functional cells of the brain classified via their associated neurotransmitters, and glial cells, such as astrocytes, microglia and oligodendrocytes, which are all critical for maintaining homeostasis and working function in the CNS [43]. iPSCs can be differentiated into several of these specialized cellular subtypes with functional characteristics that are representative of those found in the brain, such as dopaminergic neurons, cortical neurons and the aforementioned neuroglia [44].

The in vitro neuroectodermal induction of iPSCs, initiated via the dual SMAD inhibition method, results in the efficient generation of neural rosettes comprised of neuronal stem cells (NSCs) (Sox1+, Nestin+) that represent a cross-section of the neural tube (Figure 1). These NSCs can then be passaged, producing neural progenitor cells (NPCs) which can be stably maintained in culture [45]. A neuronal differentiation medium can then be applied to NPCs, which can be plated and further differentiated into more mature neuronal (Map2+, TH+, SLC18A3+) and glial cultures, including astrocytes (AQP4+, s100β+) and oligodendrocytes (NG2, Olig1/2, NBP) [46,47].

Neuronal differentiation of iPSCs provides patient-specific cells of neural lineage, opening possibilities for developing therapeutics, analysing drugs, and studying the underlying mechanisms of neurological pathologies. This is done by differentiating iPSCs into NSCs in a 2D setting which includes primitive and neural rosette-type NSCs [48,49]. Conventional 2D in vitro neural models have enabled vast knowledge enhancements regarding brain cellular subtypes, such as adhesive and migratory cellular attachment sites, formation of spontaneous networks, cell type-specific resting membrane potentials and mechanisms underlying axonal guidance [50].

On the other hand, neural differentiation of iPSCs can also be undertaken in a 3D settings. This can involve the generation of neurospheres, floating 3D NSC cultures that have been widely utilized for in-depth NPC analysis and more closely resemble the in vivo setting than 2D cultures [51,52].

Other 3D methods can utlilize artificial scaffolding or extra-cellular matrix (ECM) materials that are continuously under optimization to recapitulate the anatomical organisation of the brain [53,54]. 3D neural culture models involving cell growth using a hydrogel matrix or synthetic scaffolds are highly desirable, offering systems with intricate and easily calculable architecture with specific functional characteristics. Even though the reproducibility of these 3D models is a current challenge, newer methods that involve laser fabrication and bioprinting offer promising avenues for producing accurate and reproducible 3D in vitro neural cultures. Therefore, iPSCs have been, and will continue to be, utilized for advanced microstructured 3D scaffolds for in vitro disease modelling and for the study of neuronal functionc [55,56,57,58].

Persistent advances in the methodology used to obtain in vitro brain tissue from iPSCs led to the development of 3D brain-organoids from embryoid bodies [59]. These organoids have been demonstrated to consist of several distinctive brain regions and heterogenous tissue that can mimic the sophisticated architecture of the central nervous system [60,61]. It is worth noting, however, that as the complexity of these 3D cultures improves, so does their variability and heterogeneity. Therefore, improved methods of high content analysis will be required to determine the phenotypic characteristics of these cultures with multidimensional readouts [62].

There are many issues being investigated concerning the source, quality, stability, safety and scalability of human iPSC and derivative cell production for a variety of uses. Concerning the somatic cell source, pre-existing mutations acquired during the lifetime of the donor are more frequent in skin samples than in bone marrow. This means that very early life stage sources, for example those from umbilical cord blood banks, exhibit these potentially adverse events to a much lesser degree [63,64,65,66]. However, during the reprogramming, maintenance and scaling-up of iPSC cultures further mutations, including chromosomal rearrangements, can happen. These need to be monitored, especially in case of further clinical use [67,68,69]. The process of adaptation to the in vitro culture conditions favours some chromosomal rearrangements occurring more frequently [70]. Development of culture conditions occurrence, as well as advanced quality control methods, are an important direction of stem cell banking and the key towards clinical applicability. Major public and private entities have created human pluripotent stem cell banks with many cell lines originating from patients of different ethnic groups, yet many of them have not been consented for industrial use, and most of them have not been optimized for clinical grade applications–these are all potential hurdles to overcome if clinical applications are to be considered. The distribution of existing cell lines among ethnic groups is unbalanced, but since more nations are developing their own stem cell banks, we are gradually overcoming this ambiguity.

## 6. Specific Requirements for the In Vitro Nervous Tissue Model

While the heart muscle is a rather uniformly structured tissue, generally independent of the microanatomic region, nervous tissue brings inherent complexity stemming from the existence of various regions with a variety of cell subtypes and a multitude of functions. Thus, when cultivating cells of the nervous system in vitro, one can distuingish many types of cultures, existing in a range from mixed spontaneously differentiated and heterogenous cultures to those ones in which selection of one subtype of cells is preferred (e.g., motoric neurons, cholinergic neurons, mixed glia-neuronal cultures, astroyctes, sensoric nerons, etc). Sometimes those experiments even include chimeric interspecies cultures [71]. Another important question which brings this complexity to the next level is whether the nervous system can, if at all, be investigated focusing only on one specific cell type or region, e.g., the cerebral cortex. This is a crucial point to consider since the main function of the nervous system is to achieve a well coordinated interaction between its various regions through receiving and transmission of electrical and chemical signals.

Thus, since the physiology of the brain is rather different than that of the heart muscle, it is crucial to address all the advantages and limitations prior to starting any further development. Two dimensional cultures of nervous tissue brought numerous pioneering discoveries on cellular level, but their value in understanding higher order cellular coordination is very limited. Thus, even more than in the heart muscle, 3D cultures of nervous tissue are required for all the research aiming to elucidate physiological and pathological events occurring in interaction between cells.

## 7. Brain Organoids

While stem cells platforms based on 2D culture are being successfully used for modeling of human development and disease at cellular and molecular levels, they lack the conditions imitating spatial and temporal signaling as well as the interactions of the cells in their natural niche. These limitations of in vitro culture might be resolved by the application of biomimetic 3D solutions, especially by combining microenvironmental bioengineering with the intrinsic capacity of pluripotent stem cells to build up 3D structures [72]. This intrinsic ability of pluripotent stem cells to self-organize under 3D in vitro culture conditions into highly structured tissue patterns, opened the era of “brain organoids” [60,73]. Yoshiki Sasai and colleagues were the first to obtain highly patterned neural structures resembling muti-layered brain cortex in vitro from human pluripotent stem cells, using SFEBq (serum-free floating culture of EB-like aggregates with quick re-aggregation) protocol [73]. Further developments from the Jourgen Knoblich group brought advanced brain-like 3D in vitro structures with identified regions of cerebral cortex, retina, meninges and chordoid plexus. These 3D structures all exhibit the major stages of prenatal human brain development with functional nervous tissue cell types and cortical layer architecture, thus offering an unprecedented model for investigating human neurodevelopmental and neurodegenerative diseases [74]. Multimodal Single-Cell Analysis (single cell RT-qPCR and functional-microfluidic linked single cell RT-qPCR) of cerebral organoids cultured for more than nine months revealed a high level of neuronal and glial cell diversity as well as confirmed their functionality with identified cell-type specific responsiveness to neurotransmitters and spontaneous action potential activity [75].

Brain organoid systems appeared feasible to model early human neurodevelopment and its pathology, however they have anatomical and functional limitations which are impairing their use for studying the later developmental stages due to the lack of the correct neuronal network connectivity and vascularization. Much work in the field has been addressed towards overcoming these limitations with two parallel, but interdependent, directions: the first is focused on developing new protocols for generating replicas of multiple brain regions (development of “directed”, region specific organoids), while the second is based on constricting regulatory control of the system through bioengineering approaches.

Apart from diseases modeling, brain organoid technology can be personalized for diagnostic or therapeutic purposes if patient-specific hiPSC are applied (Figure 2) [76,77]. Whole brain (cerebral) patient-derived organoids were used to model microcephaly, macrocephaly (Sandhoff disease), periventricular heteroplasia, schizophrenia, Alzheimer Disease and other neural disorders [76,78,79]. Brain region specific organoids, e.g., forebrain to study autism spectrum disorders, or midbrain to study sporadic or idiopathic form of Parkinson’s Disease have been already obtained [80,81]. In addition, those methods are combined with a gene-editing approach with the goal to obtain “healthy/repaired” organoids by producing isogenic CRISPER/CAS9 engineered patient–derived iPSCs, as was shown for Sandhoff disease [82].

## 8. Sources of Cells for Transplantation into Nervous and Heart Tissue

Cellular therapy refers to the use of cells as medical product to treat human disorders for which other modalities of therapy either does not exist (e.g., stroke) or they are not efficient (e.g., ALS, heart decompensation). Thus, stem cell therapy has a high measurable potential in the treatment of brain and heart diseases through cell replacement and stimulation of the endogenous repair systems. Stem cells of diverse origins (embryonic stem cells, mesenchymal stem cells, induced pluripotent etc.) are all viable candidates with great potential for translation. Here we focus on two most often used stem cell types for the diseases of the brain an the heart: neural and mesenchymal stem cells.

Neural stem cells are a pluripotent cell population, expressing markers nestin and Nop2 [83], and are, thus, already inclined towards differentiation into neurons and glia. Process of forming adult cells of the nervous system, neurogenesis is a process in which neurons are generated through the division of neuronal precursors cells (NPCs) and their differentiation into neuron-specific progenitors. NPCs subsequently, over various stages of precursors, develop into fully functional and mature neurons which integrate into, and modify, existing neuronal networks. In gliogenesis, NPCs differentiate into glial progenitors, which differentiate into astrocytes, oligodendrocytes and ependymal cells.

Mesenchymal stem cells (MSC) are defined as a heterogeneous subset of stromal cells that can be easily isolated from many adult tissues and possess multilineage potential, i.e., ability to differentiate into cells of the mesodermal linage, such as adipocytes, osteocytes, chondrocytes, and myocytes [84]. Actually, the multilineage potential od MSCs allows them to differentiate into neuron-like cells, which exibit molecular and cellular characteristics of neurons. MSCs can give rise to derivatives of both ectodermal and mesodermal lineages. For example, MSCs derived from dental ligament can easily be differentiated into neurons and cardiomyocytes, opening up possibilites in treatment of neuromuscular diseases by tackling different aspects of such a complex pathophysiology [85,86,87,88,89].

## 9. Extracellular Vesicles–Desired Cellular Product on a Way towards Clinical Application

Extracellular vesicles (EVs) represent a modality for intercellular communication by acting as plasma membrane enclosed containers for a wide array of signalling molecules and ensuring transfer of biological information over long distances throughout the organism [90,91]. EVs are secreted by all types of cells and their molecular cargo reflects origin and physiological (or pathological) state of the producing cell [92,93,94]. This dichotomy is most apparent in the central nervous system (CNS), where EVs are involved in the propagation and spread of several neurodegenerative diseases [94,95]. At the same time, EVs isolated from different types of, healthy” cells can act as effective suppressors of pathological processes [93,96,97,98]. Since these particles bring therapeutic potential, it is important to develop methods for their effective labelling and follow up. Direct labelling is the simplest and most preferable method for the experiments addressing the effects of external EVs, whereas precise monitoring of behaviour and fate of cell-specific EVs within heterogeneous and 3D cultures requires more sophisticated indirect labelling techniques. We refer the readers to excellent and comprehensive reviews that provide an in depth coverage of the topic [99,100,101]. Direct EV labelling is most often performed with lipophilic dyes by inserting lipid-anchored fluorophores into the EV membranes. Many commercial dyes such as PKH26, PKH67, DiI, DiD, Dir have been developed and extensively used for EV labelling and tracking in vitro by fluorescence imaging. However, lipophilic fluorophores have several important limitations. First of all, most EV preparations isolated from different sources such as serum, or cell culture supernatants, are contaminated with lipoproteins that can also incorporate lipophilic dyes, thus leading to misinterpretation of EV uptake experiments [102]. Some lipophilic dyes also tend to aggregate, forming nanoparticles with similar size to the EVs (100 nm), that also can be taken up by the cells [102]. In addition, PKH dyes can increase EV size by enhancing clustering and aggregation [103]. These limitations can be, at least partially, overcome with the use of recently introduced Mem lipophilic dyes that did not aggregate or change the size of EVs [104]. In conclusion, although simple and straightforward, direct EV labelling with lipophilic dyes has important limitations and therefore requires careful interpretation to avoid misleading results. Indirect labelling can be achieved by CFSE (carboxyfluorescein diacetate succinimidyl ester) fluorescent dye. It is activated by esterases and covalently binds to free amines inside the cells, or vesicles. Interestingly, after indirect labelling of cells, CFSE-positive EVs were detected only in the pellets after 10,000× *g* centrifugation (corresponding to microvesicular fraction originating from plasma membrane) indicating that indirect CFSE labelling may help to distinguish between microvesicular and exosomal fractions [105]. However, large concentrations of CFSE could be necessary to obtain vesicles with sufficient fluorescence and such high dye concentrations can be detrimental to the EV-producing cells [106]. Another promising study recently used hydrophobic insertion of maleimide (Mal) into the EV membranes [107]. Other strategies are focused on RNA imaging using chemical dyes such as Alexa Fluor 488-labeled siRNA, or Cy5-siRNA, or other membrane permeable dyes that are selective for RNA that do not require conjugation, such as including E36, Styryl-TO and SYTORNAselect [108].

When coming to the topic of application of EVs, the major advantage they possess is that they can easily cross the blood brain barrier (BBB) and enter into the brain [109]. However, pharmacokinetic studies in vivo have shown that EVs can be very quickly removed from the bloodstream, with a majority of them being = entrapped in the liver and the lungs [110]. Accordingly, several groups investigated alternative delivery via minimally invasive intranasal route [111,112]. The EVs secreted by mouse macrophages were permeabilized, loaded with antioxidant enzyme catalase and applied intranasally to 6-hydroxydopamine (6-OHDA) mice [113]. Study demonstrates that EVs associated with microglia cells reduced their inflammatory activity and improved the apomorfin test results [113]. Another study compared how EVs stained with lipophilic dyes, or labelled with gold nanoparticles, distribute in the CNS after intranasal application [114]. Gold nanoparticles-marked EVs allow live observation of particle distribution in the brain by using accurate computer tomography methods. Interestingly, another study demonstrated similar distribution patterns of EVs labelled by both methods [9,114]. More importantly, EVs selectively accumulated in the affected areas of the brain. For example, after intranasal application to the 6-OHDA-treated mice (PD model), exosomes selectively accumulated in the damaged striatum areas even up to 96 h [114]. These findings confirm the potential of EVs as a therapeutic tool against various diseases and disorders of the CNS.

## 10. Cell Transplantation for Heart Ischemia

Heart failure and its direct consequences represent the leading cause of death worldwide [115]. Although heart transplantation developed substantially in the last decades, there are not enough donors which would satisfy the existing needs. Moreover, heart transplantation is a very complex and expensive procedure that afterwards require lifelong immunosuppression. The mechanism by which transplantation of stem cells into the infarcted heart leads to health improvement is not yet completely understood. The most straightforward expectation would be that transplanted stem cells form new myocardial cells with the capability to contract. However, preclinical and clinical trials revealed at least two obstacles in this theoretically simple approach: first, transplanted cells survive very briefly, so differentiation into myocardial cells is not sufficient. Second, if maturation occurs, coupling with the host myocardium is not successful. As a result, arrhythmia is a very common side effect of such an approach [116].

Preclinical studies focusing on acute infarction, e.g., with interventions within 4 weeks after the incident, reported beneficial effects [117]. On the other hand, studies which were aiming to improve condition several months after the ischemic incident were not so successful [118]. With that being said, recently, the attention has shifted from the potential of transplanted stem cell to differentiate into cardiomyocytes towards secreting factors that improve the condition of damaged myocardium [119]. Reported mechanisms include immune modulation which promotes endogenous cardiac repair [120]. Additionally, it has been shown that stem cells transplanted into the heart secrete cytokines, with rather significant anti-apoptotic effect. One of the most positive effects observed after myocardial infarction is achieved by IL-10, which improves survival and function of myocardial muscle [121].

There are many clinical trials which assessed the efficiency of stem cells for acute myocardial infarction. However, their results are rather heterogenous. Those which focused on myocardial contractility and ventricular remodeling did not find statistically significant improvement. However, significant improvements were found when a longer follow-up was taken into account, ranging from one to three years [122,123]. Most importantly, ejection fraction was regularly improved and even ventricular remodeling was shifted in a positive direction.

Even after the transplanted cells disappear, beneficial effects can be followed for months and years. Thus reduced inflammation and stimulated vascularization can be detected for a long period, reaching up to few years [124]. Thus, it became clear that, unlike pharmacologic and surgical approaches, cell therapy can stimulate endogenous tissue regeneration to reverse worsening cardiac dysfunction. Some of the most commonly reported benefits of stem cells based clinical trials are listed in the Table 1.

## 11. Specific Requirements for Further Improvement of Cell-Based Therapy of Heart Diseases

Future developments needed to boost cell-based therapy of heart diseases include nanotechnologies and bioengineered platforms, where stem cells are preconditioned to resist their implantation into a highly stressed myocardial tissue. Basically this approach consists of the development of bioactive membranes made of two integrated materials: (a) one nanofiber matrix made out of self-assembling peptides with molecule-release capacity (for growth factors such as VEGF and FGF), and (b) contained in a microscale elastomeric scaffold that provides the mechanical framework (elastic, loading) that will match the cardiac tissue mechanics. Both are essential to promote local angiogenesis in a necrotic affected tissue as well as its regeneration.

In many congenital heart diseases neonatal ventricles demonstrate a number of intrinsic pathologic modifications, including relative immaturity of the extra-cellular matrix, inappropriately low transcription factor expression and increased myocyte apoptosis, this should open the way for the evaluation of treatments associating tissue engineering with cells implants. The main mechanisms by which cell transplantation and tissue engineering can bring functional benefits in myocardial diseases is the combination of cells and scaffolds, which limit the spread of the pathologic area, preventing excessive remodeling and dilatation of the ventricle [143,144,145,146].

Emerging biomimetic technologies include 3D printing and additive manufacturing [147]. For heart healing applications, 3D-printed porous poly-caprolactone (PCL) elastomeric scaffolds represent a promising material functionalized with bio-additives such as stem cells, exosomes and angiogenic growth factors. Cardiopatch and Cardiowrap ventricular support bioprostheses were able to integrate in the damaged myocardium and the adjacent healthy heart, becoming artificial extracellular matrix that offers adequate cell niches for the homing of stem cells. These approaches contribute to the generation of Bioartificial Myocardium, offering posibility that the need for heart transplantation in the future will be reduced [127,148].

## 12. Cell Transplantation for Diseases of the Nervous System

The limited neurogenesis capacity in the brain makes neurological conditions difficult to treat. That’s why cell transplantation approach is intensively being tested for neurological diseases.

Post-ischemic acute brain injury typically peaks within 24 h of the insult, and reaches its highest point within 48 h [149]. Due to this quick onset and short duration of acute brain injury, potential neuroprotective therapies need to be administered early, i.e., within 3–6 h of the onset. This has proven to be challenging in the clinical practice. Any treatment outside of the 48 h window will offer limited neuroprotection, and could instead be mainly restorative, targeting angiogenesis, vasculogenesis, neurogenesis, and synaptogenesis [128,150]. Finding a therapeutic approach that would delay the progressive secondary neurodegeneration will also benefit stroke survivors. To date, most cell transplantation studies have been conducted on animals during acute phase of post-ischemic injury, leaving chronic time points understudied. It has already been shown that in addition to anti-inflammatory, anti-oxidative and anti-apoptotic effects, transplanted cells also secrete various factors acting neurotrophically exhibiting neuroregenerative effects [130,151].

Upon optimized dose regime and the route of administration, the use of stem cells shows benefits in both the acute and subacute phase, as well as in the chronic phase of cerebral ischemia [131,132]. Similar has been observed in other diseases with neuroinflammatory componente, like amytrophic lateral sclerosis or multiple sclerosis. Since a higher degree of neuroinflammation is present in the acute and subacute phase of cerebral ischemia, in these phases it is necessary to use higher doses (10–1200 million cells) and to choose less invasive ways of stem cell application, such as intravenous, intra-arterial, intranasal and intraperitoneal [131,133,134]. In these phases, various stem cells have shown positive effects so far. In the acute phase (1–3 days after stroke): mesenchymal stem cells (MSCs) and human mononuclear cells (MNCs), human embryonic stem cells (hESCs), human neural stem cells (hNSCs), and multipotent adult progenitor cells (MAPC) were used [131,152,153]. In the subacute phase (7 days after stroke): autologous CD34+ stem/progenitor cells and bone marrow stem cells (BMSCs) were used [131,154]. In the chronic phase (weeks, months, years) after stroke the smaller doses of stem cells were used (1–5 million cells), albeit with more invasive application methods (intracerebral and intraventricular) in order to allow greater bioavailability of injected cells near the affected brain region [128,155].

In the last two decades more than 70 clinical trials with stem cells for brain diseases have been successfully finished, but no definitive efficacy trials have been concluded. As such, there is currently still no approved cell therapy for neurological diseases. When talking about stroke, as the most common disease of the brain, various approaches have been taken thus far. Not entering into details of various type of stem cells and routes of cell delivery, all trials of Phase 1 and 2 reported safety and visibility. It is interesting to mention that one of the very first trials performed in 2005 in South Korea with 30 patients with cerebral infarct, who received IV infusion of autologous MSCs, reported a significant reduction in mortality within five years of stroke incidence compared to patients who did not receive MSC transplantation [135]. In clinical settings, the recipients of allogeneic MSCs demonstrated long-lasting or transient neurological improvement. Additionally, allogeneic MSCs infusion was associated with a short term decrease in circulating T cells and inflammatory cytokines [136]. The implantation of SB623 to the sites surrounding the subcortical stroke region was safe and accompanied by improvements in neurological recovery in 12 patients in a 2-year study [137]. At this stage, clinically confirmed beneficial effects were shown by CTX0E03 cells (hNSCs), administered one month after cerebral ischemia (a single intracerebral dose of up to 20 million cells), and SB623 (allogeneic MSCs), administered several times with 2.5, 5, and 10 million cells for a period of 6–60 months after stroke [129,131]. As the systemic inflammatory response is a major pathological component in secondary post-ischemic cell death [156], including some specific types of death, like necroptosis [157], stem cell transplantation should to be the therapy of choice to reduce neuroinflammatory effects and help stroke outcomes. Considerable numbers of clinical trials with stem cell therapy for stroke are currently underway. Clinical trials should include patient’s co-morbidities which also can affect the efficacy and effectiveness of cell therapy.

MSCs are a population of cells which can be safely harvested from patients. Due to their low immunogenicity and reported benefits, they are already being recognized as approved therapeutic product in some countries [158]. Additionally, MSCs are capable of migrating towards lesioned areas upon receiving attraction signals by certain chemokines, suggesting their potential use as vehicles for therapeutic agent’s delivery [159]. Therefore, as therapeutic agents, MSCs have multiple modes of action, including cell replacement, immunologic and metabolic properties; showing a pleiotropic activity that modify the tissues response to injuries and activate restorative mechanisms that improve organ function. Intense interchange of active cellular products between MSCs and resident cells have been proven, demonstrating the potential of MSCs secretome to achieve various paracrine effects, including immunomodulation [160]. Moreover, organelle interchange has been proven, including vesicular traffic (exosomes, microvesicles, etc), where, in addition to the vesicular cargo, MSCs inject membrane (carrying protein membrane complexes, receptors, ion channels, etc.) into host cells [161].

MSCs from the bone marrow had been widely used in clinical trials for neurological diseases. They were demonstrated to be safe but their effects were not always consistent, as preclinical studies suggested. This may be due to poor survival in disease environments and/or because inappropriate therapeutic dosage and route of delivery or inconsistent trial design [162,163,164,165].

In some studies, ALS patients treated with MSCs displayed a slight and transient decline in disease progression [138] Interestingly, postmortem evaluation of ALS patients treated with MSCs showed that a more significant number of motor neurons were preserved at the location in the spinal cord where the cells were administered, compared to other spinal sites [139]. Some of the most commonly reported benefits of stem cells based clinical trials are listed in the Table 1.

## 13. Specific Requirements for Further Improvement of Cell-Based Therapy of Brain Diseases

More than 300 papers have been published in the last 20 years reporting transplantation of cells in animal models and more than 70 clinical trials have been conducted in humans with neurological diseases with some common breakthroughs and some common obstacles. First of all, dogma that transplanted cells need to integrate and survive for a longer period is not only seen as obsolete, but in some cases is even overly stressed. Therefore, one needs to focus on cell products which are, nevertheless, being secreted in large quantities by many cell types. In addition, modification of these products can be achieved by genetic modifications of the stem cells [166]. Secreted growth factors, short sequences of RNA in various forms and still yet to be discovered components, often packed in the form of extracellular vesicles, obviously have a very strong and beneficial influence. So, it became clear that we need to focus on recognizing those beneficial products, to discover mechanism by which they improve regeneration, and then on methods how to deliver them in sufficient quantities. Apart from direct transplantation, intravascular delivery, based on positive results, deserves our attention [167]. Moreover many methodological gaps in clinical translation must be recognized. Well-designed, biomarker oriented endpoints and comparative trials are needed to address specific issues such as type of cells, cell doses, responsive phenotypes and time window of efficacy.

When thinking about side effects of cellular therapy, it is important to notice that transplantation of stem cells into brain tissue very rarely brings any significant obstacles from that side. Probably the most well defined are those linked to dyskinesia, mostly observed in transplantation to patients suffering from Parkinson’s disease. However, methods to predict which patients are more prone to those side effects have been already developed. It is interesting to notice that no serious effects coming from uncontrolled electrical activity (e.g., partial or generalized seizures) of such cells have been reported. On the other hand, common obstacles observed are a limited period of activity of such cells, with very time-limited secretion of needed molecules. Thus, the main focus is in securing longer and more substantial effects of the secretome.

## 14. Conclusions Remarks

In this review we gathered experience from the last few decades dealing with attempts to treat diseases of the heart and the brain (primarily ischemic in its nature) by using stem cells and their products (Figure 2). When we make a general overview of what has been achieved with these replacement strategies, i.e., the approach in which transplanted cells will replace lost ones in the host tissue, results are rather limited. Nevertheless, replacement therapy seems to be very promising in the case where a very specific subpopulation of neurons, in limited regions, are involved. This can be seen in positive, albeit transient, results in clinical trials including patients with Parkinson’s disease [141]. In all other cases, especially in brain ischemia (stroke) and myocardial infarction, transplanted cells can still hardly replace what has been lost. It is very interesting to notice that we expected probably much more from this approach in the heart tissue, which is, in theory, much less complex, than the neural one. However, cells which succeeded to survive in the cardiac muscle for a longer period, could hardly coordinate their activity with the rest of the healthy muscle and, most interestingly, often cause problematic arrhythmias. It is important to notice that arrhythmias in the heart muscle are a much more common problem of stem cell transplantation than uncontrolled electric activity of the transplant in the brain. In the same time, several decades of stem cells - based attempts to treat those diseases brought a huge progress in understanding of complexity of the tissue affected by pathological process. Although the ultimate goal is to discover and launch new drugs and/or procedures for human diseases, fragments of knowledge which we are collecting are without doubts constantly improving medicine.

When we take a look into the effects transplanted cells achieved with their secretome, and considering the experience in treating both the heart and the brain, this strategy emerges as a promising one. This idea has been boosted even further by the discovery of several types of extracellular vesicles which carry short sequences of RNA, peptides, growth factors, etc. In both organs, products of transplanted cells clearly influence inflammation and, in most of the cases, decrease damage with measurable effects. This is the case with neurodegenerative diseases such as ALS [168] or Alzheimer’s disease [169]. One of the probably most surprising observations, again seen in both the heart and the brain, is that those effects are often more pronounced in chronic than in acute phases. Thus, overall survival and improvement in major parameters demonstrate statistically significant differences when patients are followed after 6, 12 or 48 months [122,123,137]. How is this possible if majority of cells disappear within a few weeks after transplantation? We can think of two possible explanations: first, those cells which remain, although in small numbers, are naturally selected as those which succeed to achieve substantial positive effects. So here we obviously have an example of supreme quality ruling over quantity. Another element adding to this explanation might be that a combination of positive effects achieved by all cells, before they disappear within a few weeks after transplantation, triggers a positive chain of events which requires a lot of time to pass the threshold which is then recognized as a positive therapeutic effect. Another common point where research into the brain and the heart yielded mutual benefits for both fields is a piece of knowledge about the need for standardization of products secreted by stem cells. Standardization is not only needed in order to cause more comparable results, but also to better define routes of delivery. When this will be achieved, and many efforts are currently being undertaken in that direction, one can imagine repetitive injection of solutions with extracellular vesicles, which will improve regeneration of either neural or cardiac muscle tissue. This review could not cover all parts of this complex field, so, for example, here we did not take in consideration numerous options of genetic engineering, which offers advantages of genetically modified cells. In addition, bioengineering field based on biomaterials is progressing even faster than stem cells. By taking all this in consideration, one of the factors which slow down the progress is complexity of all these elements which requires truly multidisciplinary approach. A very wide and multidimensional perspective is needed in order to pass the threshold of success in clinical trials. To conclude, the major advice we can get from the experience collected thus far is that more standardized methods of transplantation, either with well defined populations of cells or with extracellular vesicles are needed. In addition, transplanted cells need time to bring positive effects. Clinical trials need to plan prolongued follow up of the patients and, whenever possible, account for repeated therapeutic procedures based on cells and/or their products. When such a protocol enters routine practice, we will be witnessing the final confirmation of the value of regenerative medicine in the treatment of major human diseases.

## Figures and Tables

**Table 1:** Overview of pathological entities and reported beneficial effects of cells.

Diagnoses	Requirements from Cells
Ischemic heart disease	Reduces myocardial necrosis, promotes myogenesis [119,120,121,124]
Diabetic Cardiomyopathy	Prevents apoptosis Reduces myocardial fibrosis Improves overall cardiac function [125,126]
Cardiac Tissue Engineering	Stimulates cell attachment and migration Source of biochemical factors [127]
Stroke	Reduce damage, improve recovery [128,129,130,131,132,133,134,135,136,137]
Amyotrophic lateral sclerosis	Support survival of motoric neurons [138,139]
Multiple sclerosis	Immunomodulation and decrease in demyelination [140]
Parkinson disease	Production of dopamine, reduces symptoms [141]
Spinal cord damage	Opposes anti-regenerative action of glial scar and promotes axon growth [142]

## Data Availability

Not applicable.
